# Effect of Ambrotose AO^® ^on resting and exercise-induced antioxidant capacity and oxidative stress in healthy adults

**DOI:** 10.1186/1475-2891-9-49

**Published:** 2010-11-01

**Authors:** Richard J Bloomer, Robert E Canale, Megan M Blankenship, Kelsey H Fisher-Wellman

**Affiliations:** 1 Cardiorespiratory/Metabolic Laboratory, The University of Memphis, Memphis, TN 38152, USA

## Abstract

**Background:**

The purpose of this investigation was to determine the effects of a dietary supplement (Ambrotose AO^®^) on resting and exercise-induced blood antioxidant capacity and oxidative stress in exercise-trained and untrained men and women.

**Methods:**

25 individuals (7 trained and 5 untrained men; 7 trained and 6 untrained women) received Ambrotose AO^® ^(4 capsules per day = 2 grams per day) or a placebo for 3 weeks in a random order, double blind cross-over design (with a 3 week washout period). Blood samples were collected at rest, and at 0 and 30 minutes following a graded exercise treadmill test (GXT) performed to exhaustion, both before and after each 3 week supplementation period. Samples were analyzed for Trolox Equivalent Antioxidant Capacity (TEAC), Oxygen Radical Absorbance Capacity (ORAC), malondialdehyde (MDA), hydrogen peroxide (H_2_O_2_), and nitrate/nitrite (NOx). Quality of life was assessed using the SF-12 form and exercise time to exhaustion was recorded. Resting blood samples were analyzed for complete blood count (CBC), metabolic panel, and lipid panel before and after each 3 week supplementation period. Dietary intake during the week before each exercise test was recorded.

**Results:**

No condition effects were noted for SF-12 data, for GXT time to exhaustion, or for any variable within the CBC, metabolic panel, or lipid panel (p > 0.05). Treatment with Ambrotose AO^® ^resulted in an increase in resting levels of TEAC (p = 0.02) and ORAC (p < 0.0001). No significant change was noted in resting levels of MDA, H_2_O_2_, or NOx (p > 0.05). Exercise resulted in an acute increase in TEAC, MDA, and H_2_O_2 _(p < 0.05), all which were higher at 0 minutes post exercise compared to pre exercise (p < 0.05). No condition effects were noted for exercise related data (p > 0.05), with the exception of ORAC (p = 0.0005) which was greater at 30 minutes post exercise for Ambrotose AO^® ^compared to placebo.

**Conclusion:**

Ambrotose AO^® ^at a daily dosage of 4 capsules per day increases resting blood antioxidant capacity and may enhance post exercise antioxidant capacity. However, no statistically detected difference is observed in resting or exercise-induced oxidative stress biomarkers, in quality of life, or in GXT time to exhaustion.

## Background

Oxidative stress may occur when the production of reactive oxygen and nitrogen species (RONS) overwhelms endogenous and exogenous antioxidant defenses, with the potential outcome being oxidation of large and small molecules within a variety of susceptible tissues [1]. Such findings have been reported in hundreds of investigations over the past several years, both in a rested state [2], as well as in response to aerobic [3] and anaerobic [4] exercise. While it is well accepted that a low level of RONS production is absolutely necessary to maintain normal physiological function [5], as well as to allow for exercise-induced adaptations to the endogenous antioxidant defense system [6,7], excessive RONS production may lead to the oxidation of lipids, proteins, and nucleic acids, which may ultimately impair normal cellular function [8]. For example, significant and acute elevations in RONS may impair muscle force production [9], in addition to impede exercise recovery [10]. Moreover, a chronic elevation in RONS and oxidative stress is implicated in the pathogenesis of human disease [11], and is a major factor involved in the aging process [8]. Therefore, it has been the objective of many investigators and clinicians to minimize oxidative stress levels, often done with the use of supplemental antioxidant nutrient intake.

Although antioxidant intake through whole foods, as well as low to moderate dose nutritional supplements, is generally considered to provide health-enhancing benefits, higher-dose supplemental antioxidant intake is somewhat controversial [12,13]. This controversy is related to isolated findings that antioxidant use (1000 mg vitamin C alone [12] or in combination with 400 IU vitamin E [13]) has been reported to attenuate certain adaptations that are commonly observed as a result of chronic exercise training, including enhanced parameters of insulin sensitivity [13], as well as an up-regulation in endogenous antioxidant enzymes, mitochondrial biogenesis, and endurance capacity [12]. Such findings suggest the possible need for nutritional supplements which provide a well-balanced array of antioxidant nutrients, at relatively low dosages, which may function together to provide increased antioxidant defense.

Indeed, supplemental antioxidant use is a popular practice, with an estimated 30% of Americans consuming some sort of antioxidant supplement in 2004 [14]. Based on current trends in the dietary supplement industry, it is likely that this number is actually higher today. While many popular antioxidants have been studied primarily within animal models or *in vitro *systems, and used at dosages that far exceed present recommended intake values, it is unknown what the optimal antioxidant(s) is for human supplementation.

In an effort to provide a scientifically sound and consumer friendly antioxidant supplement, many companies now include antioxidant "blends" consisting of a variety of antioxidants designed to work in conjunction with one another in redox cycling. One such product is Ambrotose AO^® ^(Mannatech, Incorporated, Coppell, TX). The Ambrotose AO^® ^supplement is a multi-component, food based, dietary supplement containing a proprietary blend of both lipid and water soluble antioxidants. In a recently published study involving a mixed sample of male and female smokers and non smokers [15], Ambrotose AO^® ^increased serum Oxygen Radical Absorbance Capacity (ORAC) by 36.6% when subjects ingested up to 8 capsules daily (500 mg per capsule = 4000 mg per 8 capsules). This study involved an open-label design, using an escalating dosing schedule of 1, 2, 4, and 8 capsules daily, over the course of a 5 week period. Using a quadratic function in an attempt to estimate the optimal dosage of Ambrotose AO^® ^to increase serum ORAC, the authors concluded that a daily dosage of 4.7 capsules per day may be ideal. Similar findings for the increase in serum ORAC were noted in another open-label study performed by Boyd and colleagues [16], when male and female smokers and non smoking subjects ingested only 1000 mg per day of Ambrotose AO^® ^for two weeks. While these data are interesting, shortcomings of these studies include the use of an open label design, the failure to include multiple biomarkers of oxidative stress, and the analysis of blood samples collected from subjects while only in a rested state.

Based on these findings, we believed that a logical follow-up to this research would be to investigate the effects of Ambrotose AO^® ^on a variety of oxidative stress biomarkers, not only at rest (as done in the previous studies), but also in response to an acute exercise stressor. Within the field of sport nutrition, the use of antioxidants (typically at high dosages) as protective agents against the stressful effects of acute exercise has received considerable attention in recent years [17,18]. Determining the effects of the Ambrotose AO^® ^supplement under such a condition is thus very timely.

Hence, the purpose of the present study was to investigate the effects of Ambrotose AO^® ^on resting and exercise-induced antioxidant capacity and oxidative stress biomarkers. In an attempt to determine if differences in responses occurred between exercise trained and untrained subjects, our sample consisted of both trained and untrained men and women. We hypothesized that Ambrotose AO^® ^supplementation would result in an increase in resting antioxidant capacity and a decrease in oxidative stress biomarkers. Additionally, it was hypothesized that acute exercise would result in an increase in oxidative stress in both conditions, with attenuation observed with the Ambrotose AO^® ^condition.

## Methods

### Subjects and Screening

Young to middle aged (20-49 yrs) exercise trained (n = 7) and untrained (n = 7) men and exercise trained (n = 7) and untrained (n = 7) women were initially recruited to participate. Eligibility was determined by completion of health history, drug and dietary supplement usage, and physical activity questionnaires. Subjects were considered to be "exercise trained" if they were engaged in regular exercise for a minimum of 4 hours per week prior to being enrolled in the study, while untrained subjects did not exercise regularly. All subjects were instructed to maintain their pre-study training program throughout the course of the study. In determining the weekly hours of exercise, the *total time *of the exercise session was accounted for and not simply the time engaged in the activity. For example, resistance training involves both work and rest intervals. In this case the cumulative time was considered and not simply the time of "work". Activities including walking, jogging, cycling, stepping, swimming, aerobics classes, and similar activities were classified as "aerobic" exercise. Activities including machine and free weight resistance training and sprinting were classified as "anaerobic" exercise. While we understand that machine and free weight resistance exercise, as well as high intensity sprint exercise, may result in adaptations to the cardiorespiratory system as well as the metabolic and skeletal muscle systems, for our classification purposes, such exercise was indicated as anaerobic. No attempt was made to classify exercise type based on percentage of heart rate response, blood lactate, etc. See Table 1 for subject descriptive characteristics. Subjects were nonsmokers, did not report any history of cardiovascular or metabolic disorders, and did not use nutritional supplements (or were willing to stop their use before and throughout the study period). Prior to participation, each subject was informed of all procedures, potential risks, and benefits associated with the study through both verbal and written form in accordance with the approved procedures of the University Institutional Review Board for Human Subjects Research. Subjects signed an informed consent form prior to being admitted as a subject.

**Table 1:** Descriptive characteristics of subjects

Variable	Trained Men (n = 7)	Untrained Men (n = 5)	Trained Women (n = 7)	Untrained Women (n = 6)
Age (yrs)	31.1 ± 5.8	32.2 ± 9.9	26.0 ± 9.1	28.8 ± 4.6
Height (cm)*	181.8 ± 9.4	176.8 ± 9.3	165.8 ± 6.7	164.3 ± 1.7
Weight (kg)*	82.1 ± 10.0	85.4 ± 8.5	61.4 ± 11.6	60.4 ± 5.2
BMI (kg·m^-2^)*	24.8 ± 2.2	27.3 ± 3.0	22.2 ± 2.7	22.3 ± 2.0
Body fat (%)†*	11.2 ± 5.5	17.5 ± 5.4	18.7 ± 5.6	23.8 ± 5.7
Waist (cm)*	84.8 ± 5.0	89.7 ± 9.4	68.8 ± 4.6	70.6 ± 3.7
Hip (cm)*	102.6 ± 6.0	104.2 ± 4.7	95.9 ± 7.6	96.3 ± 4.9
Waist:Hip*	0.83 ± 0.03	0.86 ± 0.08	0.71 ± 0.04	0.73 ± 0.03
Resting HR (bpm)	57.1 ± 6.7	62.6 ± 8.6	60.0 ± 14.4	71.5 ± 10.1
Resting SBP (mmHg)*	121.1 ± 9.6	125.6 ± 12.1	114.7 ± 4.1	112.3 ± 7.6
Resting DBP (mmHg)*	80.3 ± 8.3	85.6 ± 5.4	72.6 ± 6.4	77.0 ± 3.7
Years Anaerobic Exercise†	10.1 ± 3.7	0.8 ± 1.2	8.4 ± 9.8	0.5 ± 0.8
Hours per week Anaerobic Exercise†	3.3 ± 0.9	0.4 ± 1.2	4.5 ± 2.9	0.3 ± 0.5
Years Aerobic Exercise†	9.1 ± 9.7	2.6 ± 4.4	4.1 ± 3.7	1.6 ± 5.2
Hours per week Aerobic Exercise†	4.0 ± 3.9	0.7 ± 0.6	4.6 ± 3.6	0.6 ± 0.6

Values are mean ± SD.

† Training status effect: Body fat (p = 0.02); Years Anaerobic Exercise (p = 0.001); Hours per week Anaerobic Exercise (p = 0.0001); Years Aerobic Exercise (p = 0.02); Hours per week Aerobic Exercise (p = 0.004)

* Sex effect: Height (p < 0.0001); Weight (p < 0.0001); BMI (p = 0.0009); Body fat (p = 0.007); Waist (p < 0.0001); Hip (p = 0.008); Waist:Hip (p < 0.0001); Resting SBP (p = 0.01); Resting DBP (p = 0.004)

No other statistically significant differences were noted (p > 0.05).

### Measurements

Subjects' height (via stadiometer), weight (via electronic scale), and body composition (via a 7 site skinfold test and calculation using the Siri equation) was measured. Heart rate (via 60 second palpation) and blood pressure (via auscultation) were recorded following a 10 minute period of quiet rest. A maximal graded exercise test (GXT) was conducted using a treadmill, and subjects continued until exhaustion.

### Graded Exercise Test

Following each 21 day period of Ambrotose AO^® ^and placebo intake, subjects reported to the lab in the morning to perform a GXT on a treadmill. A GXT was chosen for the exercise stressor, as this test involves both an aerobic and anaerobic component. Both forms of exercise have been reported to result in an acute increase in oxidative stress [3,4], possibly due to a combination of factors such as increased oxygen consumption, catecholamine auto-oxidation, ischemia-reperfusion, prostanoid metabolism, xanthine oxidase activity, inflammation, and malfunctions in calcium handling [4]. In an attempt to maintain consistency in testing, a script was read to each subject prior to performing the GXT. The protocol involved an increase in intensity every 2 minutes in the following manner: min 1-2, 3.0 mph, 0%; min 3-4, 3.5 mph, 0%; min 5-6, 4.0 mph, 0%; min 7-8, 4.5 mph, 0%; min 9-10, 5.0 mph, 0%; min 11-12, 5.0 mph, 5%; min 13-14, 5.5 mph, 5%; min 15-16, 5.5 mph, 7.5%; min 17-18, 6.0 mph, 7.5%; min 19-20, 6.0 mph, 10%; min 21-22, 6.5 mph, 10%; min 23-24, 6.5 mph, 12.5%; min 25-26, 7.0 mph, 12.5%. Using the above protocol, for most subjects minutes 1-6 served as an aerobic warm-up, minutes 7-12 served as an initial aerobic/anaerobic challenge, and times after 12 minutes served to anaerobically stress the subject until they reached exhaustion. No subject exceeded 25 minutes of testing. This identical protocol was administered following each of the 3 week supplementation periods, with the exact script read prior to each GXT. Before and during the GXT, heart rate was continuously monitored via electrocardiograph (ECG) tracings using a SensorMedics Max-1™ ECG unit and the Borg scale of exertion was used to allow subjects to indicate their level of perceived work. Total exercise time was also recorded. Although subjects performed the GXT in the morning following an overnight fast, they were allowed to drink water *ad libitum *before and following the GXT.

While we were primarily interested in generating an increase in RONS production by having subjects perform the GXT, rather than in monitored hemodynamics, the blood pressure response to exercise was not measured. Hence, we are unable to provide data related to the rate pressure product during exercise. Moreover, we did not collect expired gases during exercise, as doing so sometimes limits subjects' effort due to difficulty in breathing into a facemask with the inability to breathe through the nose. Therefore, we are unable to provide data related to VO_2 max _during exercise. Our failure to include the above measures may be considered by some to be limitations of this work.

At the conclusion of the GXT, a full explanation of dietary data recording was provided to subjects, along with data collection forms. An overview of all study procedures was also provided. Subjects were then assigned their initial condition (Ambrotose AO^® ^or placebo), instructed on how to take the capsules, and scheduled for their remaining laboratory visits.

### Supplementation

**Figure 1:**
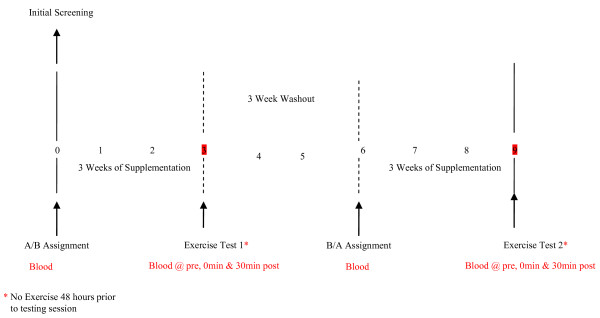
**Timeline of study to investigate the effect of Ambrotose AO^® ^on resting and exercise-induced antioxidant capacity and oxidative stress in healthy adults**.

The study design involved a random order, cross-over assignment to Ambrotose AO^® ^or placebo in a double blind manner. A schematic overview of the study timeline is presented in Figure 1. Subjects ingested 4 capsules per day of Ambrotose AO^® ^or placebo with meals (2 capsules in the morning and 2 capsules in the evening) for a total of 21 days, with a 21 day wash out period between conditions. Both the Ambrotose AO^® ^and placebo capsules (cellulose) were provided by Mannatech, Incorporated (Coppell, TX), and were virtually identical in appearance and texture. Each capsule of Ambrotose AO^® ^contained 18 mg vitamin E as mixed tocopherols; 113 mg of an antioxidant blend (quercetin dihydrate; grape skin extract; green tea extract; *Terminalia ferdinandiana *[Australian bush plum powder], 331 mg of a proprietary blend of plant polysaccharides and fruits and vegetables powders (aloe vera inner leaf gel, gum acacia, xanthan gum, gum tragacanth, gum ghatti, broccoli, Brussels sprouts, cabbage, carrot, cauliflower, garlic, kale, onion, tomato, turnip, papaya and pineapple). For both conditions, capsules were distributed to subjects by research assistants in unlabeled bottles in amounts greater than needed for supplementation. Capsule counts upon bottle return allowed for estimation of compliance to intake.

### SF-12 Questionnaire

Subjects were asked to complete a questionnaire pertaining to their overall mental and physical health status (SF-12v2; QualityMetric, Inc.). The questionnaire was delivered using a computer based program and scoring was performed using automated software immediately following completion of the questionnaire.

### Blood Sampling

Venous blood samples (~20 mL) were collected from subjects' forearm via needle and Vacutainer™ before and following each 21 day period of supplementation with Ambrotose AO^® ^and placebo (blood collections occurred on days 1 and 22). Measurements of all antioxidant and oxidative stress variables were done at rest (following a 10 minute quiet rest), immediately after the GXT, and 30 minutes after the GXT. For resting samples only (pre and post intervention), a portion of blood was processed accordingly and sent to Laboratory Corporation of America for analysis of complete blood count, metabolic panel, and lipid panel within 24 hours of collection using automated clinical analyzers. Samples collected in containers with no additive were allowed to clot for 30 minutes at room temperature and were then centrifuged at 2000 g at 4°C to obtain serum. Samples collected in containers with EDTA were immediately centrifuged at 2000 g at 4°C to obtain plasma. Following centrifugation, the serum/plasma was immediately stored in multiple aliquots in an ultra-low freezer until analyzed for antioxidant and oxidative stress variables.

### Biochemistry: Antioxidant and Oxidative Stress Variables

The following variables representing antioxidant capacity and oxidative stress were chosen based on their use within the exercise science/nutrition literature, in particular related to oxidative stress markers [19]. A second consideration was their relative ease of analysis, in that replication of this work would be possible by most laboratories. A limitation of this work is the exclusion of protein and DNA specific markers of oxidative stress such as protein carbonyls and 8-hydroxydeoxyguanosine, as well as the exclusion of individual enzymatic and non-enzymatic antioxidants.

Antioxidant capacity was analyzed in serum using the Trolox-Equivalent Antioxidant Capacity (TEAC) assay using procedures outlined by the reagent provider (Sigma Chemical; St. Louis, MO). Antioxidant capacity was also analyzed in serum (following a 750 fold dilution) using the Oxygen Radical Absorbance Capacity (ORAC) assay using procedures outlined by the reagent provider (Zen-Bio, Inc.; Research Triangle Park, NC). It should be noted that several methods are available to assess the **"**total" antioxidant capacity of blood. These include TEAC (which appears primarily influenced by urate) and ORAC, as well as the ferric reducing ability of plasma (FRAP) assay and the total radical-trapping antioxidant parameter (TRAP) assay. Of these, ORAC and FRAP have been noted to be well-correlated, while TEAC is not correlated with ORAC or FRAP, and may underestimate antioxidant capacity [20]. Therefore, as with the oxidative stress biomarkers, we chose to include more than one antioxidant capacity marker, as has been suggested previously [21].

Malondialdehyde (MDA) was analyzed in plasma using a commercially available colorimetric assay (Northwest Life Science Specialties; Vancouver, WA), using the modified method described by Jentzsch et al. [22]. Hydrogen peroxide (H_2_O_2_) was analyzed in plasma using the Amplex Red reagent method as described by the manufacturer (Molecular Probes; Invitrogen Detection Technologies, Eugene, OR). Nitric oxide (NOx) was estimated using the nitrate/nitrite assay procedure as described by the manufacturer (Caymen Chemical; Ann Arbor, MI). All assays were performed on first thaw.

### Dietary Intake and Physical Activity

All subjects were instructed to maintain their normal diet, without attempts to increase or decrease antioxidant nutrient intake. Subjects recorded their food and beverage intake during the seven days prior to each exercise test day. Nutritional records were analyzed for total calories, protein, carbohydrate, fat, and a variety of micronutrients (Food Processor SQL, version 9.9, ESHA Research, Salem, OR). Subjects were given specific instructions regarding abstinence from alcohol consumption during the 48 hours immediately preceding the test days. They were instructed to maintain their normal physical activity, with the exception of refraining from strenuous physical activity during the 48 hours preceding each test day.

### Statistical Analysis

For the main analysis, all outcomes measures were analyzed using a condition × time × training status × sex repeated measures analysis of variance (ANOVA). All resting blood measures (antioxidant capacity, oxidative stress, complete blood count, metabolic panel, lipid panel), in addition to SF-12 data, were analyzed using a condition × time (pre and post intervention) × training status × sex ANOVA. Exercise time to exhaustion data were analyzed using a condition × training status × sex ANOVA. Single degree of freedom contrasts, a form of post hoc testing which explicitly compares the effect of the independent variable on the outcome variables, were performed where appropriate. Dietary and supplement compliance data were analyzed using a t-test. Effect size calculations were performed using Cohen's *d*. All analyses were performed using JMP statistical software (version 4.0.3, SAS Institute, Cary, NC). Statistical significance was set at p ≤ 0.05. The data are presented as mean ± SEM, except for subject descriptive characteristics (mean ± SD).

## Results

### Overview and Compliance

Although 28 subjects were initially enrolled in the study, two untrained men dropped out during the first 3 weeks of the study due to lack of interest, and one untrained woman was dropped during the final 2 weeks of the study due to an acute illness (minor nosebleeds), which was determined to be unrelated to the study protocol. Therefore, only 25 subjects were included in the analysis (see Table 1 for descriptive characteristics). Regarding compliance to capsule intake, subjects were 90% compliant to Ambrotose AO^® ^capsules and 93% compliant to placebo capsules, with no statistical difference noted between conditions (p > 0.05). Compared with untrained subjects, however, trained subjects were significantly more compliant (p < 0.05). Data are presented in Table 2.

**Table 2:** Capsule compliance, quality of life (SF-12) and graded exercise test (GXT) time to exhaustion data of men (A) and women (B) before and following three weeks of supplementation with Ambrotose AO^® ^at a dosage of 4 capsules per day and placebo (cross-over design with a three week washout between conditions).

A								
**Variable**	**Trained Men Ambrotose AO^® ^Pre**	**Trained Men Ambrotose AO^® ^Post**	**Trained Men Placebo Pre**	**Trained Men Placebo Post**	**Untrained Men Ambrotose AO^® ^Pre**	**Untrained Men Ambrotose AO^® ^Post**	**Untrained Men Placebo Pre**	**Untrained Men Placebo Post**

% Capsule Compliance†	NA	91.9 ± 5.5	NA	98.6 ± 1.1	NA	88.6 ± 5.5	NA	89.8 ± 7.0
Physical Health†	55.4 ± 2.5	56.7 ± 1.1	57.9 ± 1.2	56.9 ± 0.8	51.2 ± 4.6	48.4 ± 3.5	54.0 ± 1.2	55.0 ± 1.6
Mental Health**	55.3 ± 1.7	54.7 ± 2.3	55.1 ± 2.7	55.1 ± 1.9	48.5 ± 6.5	52.4 ± 5.3	49.2 ± 3.8	49.4 ± 3.6
Exercise Time (sec)†*	NA	1252 ± 45	NA	1275 ± 47	NA	956 ± 65	NA	989 ± 88

B								

**Variable**	**Trained Women Ambrotose AO^® ^Pre**	**Trained Women Ambrotose AO^® ^Post**	**Trained Women Placebo Pre**	**Trained Women Placebo Post**	**Untrained Women Ambrotose AO^® ^Pre**	**Untrained Women Ambrotose AO^® ^Post**	**Untrained Women Placebo Pre**	**Untrained Women Placebo Post**

% Capsule Compliance†	NA	96.3 ± 1.3	NA	93.2 ± 2.6	NA	82.5 ± 8.7	NA	89.8 ± 5.5
Physical Health†	56.6 ± 1.0	55.3 ± 1.8	57.9 ± 1.7	56.3 ± 2.3	54.7 ± 1.3	54.7 ± 2.1	54.2 ± 2.4	53.7 ± 1.6
Mental Health**	50.3 ± 2.6	49.9 ± 2.5	49.7 ± 3.0	51.6 ± 3.0	54.0 ± 2.2	53.2 ± 3.8	51.4 ± 2.1	53.7 ± 1.5
Exercise Time (sec)†*	NA	1062 ± 59	NA	1064 ± 66	NA	839 ± 80	NA	853 ± 76

Values are mean ± SEM.

† Training status effect: Capsule Compliance (p = 0.04); Physical Health (p = 0.0008); Exercise Time (p < 0.0001)

* Sex effect: Exercise Time (p = 0.001)

** Sex × training status effect: Mental Health (p = 0.02)

No other statistically significant differences were noted (p > 0.05).

### SF-12 Data

No condition differences were noted for either mental or physical SF-12 data (p > 0.05). However, a difference was noted for physical health between trained and untrained subjects (p < 0.05). Data are presented in Table 2.

### Exercise Test Data

No condition differences were noted for GXT time to exhaustion (p > 0.05). However, a difference was noted between men and women and between trained and untrained subjects (p < 0.05). Data are presented in Table 2.

### Complete Blood Count, Metabolic Panel, Lipid Panel Data

No condition differences were noted for complete blood count (Table 3), metabolic panel (Table 4), or lipid panel (Table 5) (p > 0.05). However, several differences were noted between men and women and between trained and untrained subjects for these variables (p < 0.05), as can be seen in Tables 3, 4, and 5.

**Table 3:** Complete blood count data of men (A) and women (B) before and following three weeks of supplementation with Ambrotose AO^® ^at a dosage of 4 capsules per day and placebo (cross-over design with a three week washout between conditions).

A								
**Variable**	**Trained Men Ambrotose AO^® ^Pre**	**Trained Men Ambrotose AO^® ^Post**	**Trained Men Placebo Pre**	**Trained Men Placebo Post**	**Untrained Men Ambrotose AO^® ^Pre**	**Untrained Men Ambrotose AO^® ^Post**	**Untrained Men Placebo Pre**	**Untrained Men Placebo Post**

WBC (10^3 ^μL)†	4.5 ± 0.1	4.5 ± 0.2	5.1 ± 0.6	4.9 ± 0.3	5.7 ± 0.5	6.0 ± 0.4	5.9 ± 0.5	5.6 ± 0.5
RBC (10^6 ^μL)*	4.9 ± 0.1	4.7 ± 0.1	4.9 ± 0.1	4.8 ± 0.1	4.7 ± 0.2	4.6 ± 0.2	4.6 ± 0.3	4.6 ± 0.2
Hemoglobin (g·dL^-1^)*	14.7 ± 0.3	13.3 ± 0.5	14.8 ± 0.4	14.6 ± 0.3	14.8 ± 0.5	14.6 ± 0.5	14.6 ± 0.6	14.4 ± 0.4
Hematocrit (%)†*	43.3 ± 0.8	41.8 ± 1.1	43.5 ± 1.0	42.3 ± 0.5	43.0 ± 1.0	41.9 ± 1.2	42.2 ± 2.0	41.2 ± 1.3
MCV (fL)**	88.7 ± 2.2	89.0 ± 2.5	88.3 ± 2.3	88.3 ± 2.2	91.2 ± 2.6	90.8 ± 2.3	91.6 ± 2.2	91.0 ± 2.5
MCH (pg)**	30.2 ± 1.0	30.5 ± 1.0	29.9 ± 1.0	30.3 ± 0.9	31.4 ± 1.0	31.7 ± 1.0	31.7 ± 1.0	31.9 ± 1.1
MCHC (g·dL^-1^)**	34.0 ± 0.4	34.2 ± 0.2	33.9 ± 0.4	34.4 ± 0.4	34.5 ± 0.4	34.9 ± 0.3	34.5 ± 0.3	35.0 ± 0.4
RDW (%)**	13.8 ± 0.4	13.2 ± 0.3	13.6 ± 0.3	13.5 ± 0.3	13.0 ± 0.2	13.1 ± 0.2	12.9 ± 0.2	12.9 ± 0.2
Platelets (10^3 ^μL)*	208.0 ± 16.7	204.0 ± 13.9	214.4 ± 16.1	211.4 ± 21.7	217.6 ± 20.4	203.2 ± 21.0	208.0 ± 24.5	209.6 ± 24.3
Neutrophils (%)	51.1 ± 4.5	50.0 ± 3.4	52.7 ± 4.9	51.0 ± 4.6	54.2 ± 2.6	58.0 ± 4.6	52.8 ± 2.1	53.6 ± 3.6
Lymphocytes (%)	36.0 ± 3.8	37.4 ± 3.2	36.0 ± 4.4	36.4 ± 4.3	32.6 ± 2.1	30.2 ± 4.1	34.2 ± 1.3	33.5 ± 2.9
Monocytes (%)†	7.7 ± 0.6	8.6 ± 0.8	7.6 ± 0.6	8.3 ± 0.5	7.4 ± 0.9	7.2 ± 1.2	7.0 ± 0.5	7.1 ± 0.9
Eosinophils (%)*	4.9 ± 1.8	3.7 ± 0.8	3.1 ± 0.7	3.9 ± 0.6	5.2 ± 1.6	4.0 ± 1.0	5.8 ± 1.8	5.3 ± 1.8
Basophils (%)	0.3 ± 0.2	0.3 ± 0.2	0.6 ± 0.2	0.4 ± 0.2	0.6 ± 0.2	0.6 ± 0.2	0.2 ± 0.2	0.5 ± 0.2

B								

**Variable**	**Trained Women Ambrotose AO^® ^Pre**	**Trained Women Ambrotose AO^® ^Post**	**Trained Women Placebo Pre**	**Trained Women Placebo Post**	**Untrained Women Ambrotose AO^® ^Pre**	**Untrained Women Ambrotose AO^® ^Post**	**Untrained Women Placebo Pre**	**Untrained Women Placebo Post**

WBC (10^3 ^μL)†	5.1 ± 1.0	5.2 ± 0.7	4.7 ± 0.4	5.3 ± 0.7	5.6 ± 0.5	4.9 ± 0.3	5.0 ± 0.4	6.0 ± 0.8
RBC (10^6 ^μL)*	4.2 ± 0.1	4.1 ± 0.1	4.2 ± 0.1	4.2 ± 0.1	4.2 ± 0.1	4.1 ± 0.1	4.2 ± 0.1	4.2 ± 0.2
Hemoglobin (g·dL^-1^)*	13.4 ± 0.4	12.8 ± 0.3	13.2 ± 0.1	13.2 ± 0.2	12.5 ± 0.3	12.1 ± 0.4	12.3 ± 0.5	12.4 ± 0.6
Hematocrit (%)†*	38.8 ± 1.0	37.1 ± 0.9	38.6 ± 0.4	37.9 ± 0.6	36.9 ± 0.8	35.0 ± 0.8	36.6 ± 1.4	36.5 ± 1.5
MCV (fL)**	91.9 ± 1.7	91.4 ± 1.8	91.4 ± 2.0	90.8 ± 2.0	87.1 ± 1.2	85.8 ± 0.7	86.5 ± 0.9	86.2 ± 0.9
MCH (pg)**	31.7 ± 0.7	31.6 ± 0.7	31.1 ± 0.7	31.5 ± 0.7	29.6 ± 0.4	29.7 ± 0.4	29.0 ± 0.4	29.3 ± 0.6
MCHC (g·dL^-1^)**	34.6 ± 0.2	34.6 ± 0.2	34.1 ± 0.2	34.7 ± 0.2	33.9 ± 0.2	34.6 ± 0.3	33.6 ± 0.2	33.9 ± 0.4
RDW (%)**	13.1 ± 0.3	13.3 ± 0.3	13.2 ± 0.2	13.5 ± 0.3	13.3 ± 0.5	13.6 ± 0.6	13.7 ± 0.6	13.7 ± 0.6
Platelets (10^3 ^μL)*	234.3 ± 20.0	229.1 ± 17.1	246.1 ± 23.6	236.1 ± 18.8	256.9 ± 29.9	259.2 ± 25.4	254.8 ± 35.6	267.0 ± 34.2
Neutrophils (%)	54.1 ± 5.5	56.3 ± 4.8	57.7 ± 4.7	58.8 ± 3.9	57.3 ± 3.5	56.0 ± 2.0	53.8 ± 3.2	58.3 ± 3.4
Lymphocytes (%)	34.3 ± 4.9	31.0 ± 4.0	30.6 ± 4.4	29.3 ± 3.2	34.3 ± 3.5	35.8 ± 1.8	36.5 ± 2.6	32.5 ± 3.0
Monocytes (%)†	8.0 ± 0.9	9.0 ± 1.0	7.3 ± 0.4	8.4 ± 0.6	6.1 ± 0.5	6.0 ± 0.5	6.2 ± 0.7	5.5 ± 0.8
Eosinophils (%)*	3.0 ± 0.5	3.3 ± 0.9	3.4 ± 0.6	3.0 ± 0.6	1.7 ± 0.5	1.8 ± 0.2	3.0 ± 0.6	2.7 ± 0.5
Basophils (%)	0.6 ± 0.2	0.4 ± 0.2	1.0 ± 0.0	0.5 ± 0.2	0.6 ± 0.2	0.3 ± 0.2	0.5 ± 0.2	0.5 ± 0.2

Values are mean ± SEM.

† Training status effect: WBC (p = 0.02); Hematocrit (p = 0.02); Monocytes (p = 0.0001)

* Sex effect: RBC (p < 0.0001); Hemoglobin (p < 0.0001); Hematocrit (p < 0.0001); Platelets (p = 0.004); Eosinophils (p = 0.002)

** Sex × training status effect: MVC (p = 0.0002); MCH (p < 0.0001); MCHC (p = 0.008); RDW (p = 0.03)

No other statistically significant differences were noted (p > 0.05).

**Table 4:** Comprehensive metabolic panel data of men (A) and women (B) before and following three weeks of supplementation with Ambrotose AO^® ^at a dosage of 4 capsules per day and placebo (cross-over design with a three week washout between conditions).

A								
**Variable**	**Trained Men Ambrotose AO^® ^Pre**	**Trained Men Ambrotose AO^® ^Post**	**Trained Men Placebo Pre**	**Trained Men Placebo Post**	**Untrained Men Ambrotose AO^® ^Pre**	**Untrained Men Ambrotose AO^® ^Post**	**Untrained Men Placebo Pre**	**Untrained Men Placebo Post**

Glucose (mg·dL^-1^)**	84.3 ± 3.8	87.0 ± 4.6	87.4 ± 3.6	83.9 ± 2.3	90.4 ± 3.4	95.8 ± 5.4	99.6 ± 9.0	96.2 ± 9.1
BUN (mg·dL^-1^)†*	16.9 ± 1.9	16.7 ± 1.0	16.7 ± 1.6	17.9 ± 1.8	15.0 ± 2.3	15.8 ± 2.5	14.6 ± 1.0	13.4 ± 1.2
Creatinine (mg·dL^-1^)†*	1.1 ± 0.0	1.1 ± 0.0	1.2 ± 0.0	1.1 ± 0.0	1.0 ± 0.1	1.0 ± 0.1	1.1 ± 0.1	1.1 ± 0.1
BUN/Creatinine	15.6 ± 2.0	15.4 ± 1.4	14.6 ± 1.3	16.0 ± 1.7	15.4 ± 3.2	16.2 ± 3.7	13.6 ± 1.9	12.2 ± 1.4
Sodium (mmol·L^-1^)	138.4 ± 0.3	139.9 ± 0.6	139.3 ± 0.6	138.3 ± 0.8	137.2 ± 0.4	137.4 ± 0.6	138.8 ± 1.1	137.6 ± 1.3
Potassium (mmol·L^-1^)	4.5 ± 0.2	4.3 ± 0.2	4.3 ± 0.2	4.3 ± 0.2	4.4 ± 0.1	4.2 ± 0.1	4.1 ± 0.1	4.3 ± 0.1
Chloride (mmol·L^-1^)*	102.0 ± 0.4	103.1 ± 0.3	101.7 ± 0.9	101.9 ± 0.5	101.6 ± 0.9	101.6 ± 0.8	102.6 ± 1.4	102.2 ± 1.3
CO_2 _(mmol·L^-1^)*	26.7 ± 0.7	26.9 ± 0.6	26.7 ± 0.5	26.4 ± 0.7	26.0 ± 0.7	24.8 ± 0.7	26.2 ± 0.7	25.4 ± 0.5
Calcium (mg·dL^-1^)*	9.2 ± 0.2	9.3 ± 0.1	9.4 ± 0.1	9.4 ± 0.1	9.4 ± 0.1	9.2 ± 0.1	9.2 ± 0.1	9.3 ± 0.1
Protein (g·dL^-1^)†*	6.7 ± 0.1	6.6 ± 0.1	6.8 ± 0.1	6.8 ± 0.1	7.0 ± 0.2	6.9 ± 0.2	6.8 ± 0.1	6.7 ± 0.2
Albumin (g·dL^-1^)	4.3 ± 0.1	4.3 ± 0.1	4.4 ± 0.2	4.3 ± 0.1	4.5 ± 0.1	4.4 ± 0.2	4.3 ± 0.0	4.1 ± 0.1
Globulin (g·dL^-1^)†*	2.4 ± 0.2	2.4 ± 0.2	2.5 ± 0.2	2.5 ± 0.1	2.5 ± 0.1	2.5 ± 0.1	2.4 ± 0.1	2.6 ± 0.1
A/G Ratio†*	1.8 ± 0.1	1.9 ± 0.2	1.8 ± 0.1	1.7 ± 0.1	1.8 ± 0.1	1.8 ± 0.1	1.8 ± 0.1	1.6 ± 0.1
Bilirubin (mg·dL^-1^)**	0.7 ± 0.2	0.6 ± 0.2	0.7 ± 0.2	0.5 ± 0.1	0.8 ± 0.1	0.7 ± 0.1	0.8 ± 0.2	0.9 ± 0.2
Alk Phos (IU·L^-1^)*	71.7 ± 6.7	71.6 ± 6.7	75.3 ± 6.7	73.4 ± 6.2	68.2 ± 9.6	68.0 ± 9.0	69.0 ± 8.9	65.6 ± 10.3
AST (SGOT) (IU·L^-1^)**	25.6 ± 1.8	24.9 ± 3.4	27.1 ± 2.9	25.0 ± 1.6	25.2 ± 2.9	30.8 ± 3.5	30.6 ± 3.6	30.6 ± 4.3
ALT (SGPT) (IU·L^-1^)**	23.7 ± 2.1	26.0 ± 3.0	26.4 ± 3.2	27.1 ± 4.4	25.6 ± 7.0	33.2 ± 11.8	39.4 ± 11.9	33.0 ± 7.7

B								

**Variable**	**Trained Women Ambrotose AO^® ^Pre**	**Trained Women Ambrotose AO^® ^Post**	**Trained Women Placebo Pre**	**Trained Women Placebo Post**	**Untrained Women Ambrotose AO^® ^Pre**	**Untrained Women Ambrotose AO^® ^Post**	**Untrained Women Placebo Pre**	**Untrained Women Placebo Post**

Glucose (mg·dL^-1^)**	86.1 ± 3.3	86.7 ± 1.9	88.1 ± 2.5	97.1 ± 10.3	84.6 ± 1.9	84.8 ± 2.5	86.0 ± 1.6	82.8 ± 2.7
BUN (mg·dL^-1^)†*	14.9 ± 1.9	15.7 ± 1.9	15.6 ± 2.2	17.1 ± 2.2	11.0 ± 1.3	11.2 ± 1.4	12.5 ± 1.9	12.5 ± 2.1
Creatinine (mg·dL^-1^)†*	1.0 ± 0.0	0.9 ± 0.0	1.0 ± 0.1	0.9 ± 0.1	0.8 ± 0.0	0.8 ± 0.1	0.8 ± 0.1	0.8 ± 0.0
BUN/Creatinine	15.4 ± 2.1	16.6 ± 2.2	16.7 ± 2.7	19.1 ± 2.9	13.7 ± 1.6	14.5 ± 2.3	15.2 ± 2.5	15.3 ± 2.9
Sodium (mmol·L^-1^)	138.9 ± 0.7	138.4 ± 0.8	139.0 ± 0.9	138.0 ± 0.6	139.3 ± 0.8	138.3 ± 0.9	139.0 ± 0.9	138.3 ± 0.6
Potassium (mmol·L^-1^)	4.1 ± 0.1	4.1 ± 0.2	4.2 ± 0.1	4.3 ± 0.1	4.3 ± 0.1	4.1 ± 0.1	4.1 ± 0.1	4.4 ± 0.2
Chloride (mmol·L^-1^)*	103.1 ± 0.5	103.4 ± 0.8	103.6 ± 0.8	103.4 ± 1.0	102.6 ± 1.2	103.0 ± 0.9	103.7 ± 0.6	103.0 ± 0.5
CO_2 _(mmol·L^-1^)*	23.7 ± 0.8	24.4 ± 1.2	25.1 ± 1.0	24.4 ± 0.5	24.1 ± 1.1	24.0 ± 0.4	24.0 ± 0.3	24.0 ± 0.4
Calcium (mg·dL^-1^)*	9.6 ± 0.2	9.5 ± 0.2	9.5 ± 0.1	9.6 ± 0.1	9.4 ± 0.1	9.3 ± 0.1	9.4 ± 0.1	9.5 ± 0.1
Protein (g·dL^-1^)†*	7.0 ± 0.2	6.7 ± 0.2	6.9 ± 0.1	6.7 ± 0.2	7.1 ± 0.2	7.2 ± 0.1	7.4 ± 0.2	7.4 ± 0.2
Albumin (g·dL^-1^)	4.5 ± 0.1	4.2 ± 0.1	4.3 ± 0.1	4.2 ± 0.1	4.4 ± 0.1	4.3 ± 0.1	4.5 ± 0.1	4.4 ± 0.1
Globulin (g·dL^-1^)†*	2.5 ± 0.1	2.5 ± 0.1	2.6 ± 0.1	2.5 ± 0.1	2.8 ± 0.2	2.9 ± 0.1	2.9 ± 0.2	3.0 ± 0.2
A/G Ratio†*	1.8 ± 0.1	1.7 ± 0.0	1.7 ± 0.1	1.7 ± 0.1	1.6 ± 0.1	1.5 ± 0.1	1.6 ± 0.1	1.5 ± 0.1
Bilirubin (mg·dL^-1^)**	0.6 ± 0.1	0.5 ± 0.1	0.6 ± 0.1	0.7 ± 0.2	0.4 ± 0.1	0.5 ± 0.0	0.4 ± 0.1	0.4 ± 0.1
Alk Phos (IU·L^-1^)*	55.4 ± 6.5	51.9 ± 5.1	56.0 ± 4.5	49.6 ± 4.6	53.1 ± 6.9	53.7 ± 5.2	56.2 ± 6.8	58.3 ± 7.8
AST (SGOT) (IU·L^-1^)**	25.4 ± 2.8	29.3 ± 5.8	20.3 ± 2.1	23.3 ± 1.1	19.6 ± 2.5	18.8 ± 1.6	21.3 ± 1.7	23.2 ± 3.2
ALT (SGPT) (IU·L^-1^)**	22.1 ± 3.1	24.3 ± 4.7	19.9 ± 2.4	19.1 ± 1.5	16.3 ± 3.6	13.7 ± 2.2	14.0 ± 1.2	18.8 ± 3.8

Values are mean ± SEM.

† Training status effect: BUN (p = 0.002); Creatinine (p < 0.0001); Protein (p = 0.001); Globulin (p = 0.003); A/G Ratio (p = 0.04)

* Sex effect: BUN (p = 0.05); Creatinine (p = 0.002); Chloride (p = 0.008); CO_2 _(p < 0.0001); Calcium (p = 0.03); Protein (p = 0.002); Globulin (p = 0.006); A/G Ratio (p = 0.05); Alk Phos (p < 0.0001)

** Sex × training status effect: Glucose (p = 0.005); Bilirubin (p = 0.01); AST (p = 0.02); ALT (p = 0.01)

No other statistically significant differences were noted (p > 0.05).

**Table 5:** Blood lipid data of men (A) and women (B) before and following three weeks of supplementation with Ambrotose AO^® ^at a dosage of 4 capsules per day and placebo (cross-over design with a three week washout between conditions).

A								
**Variable**	**Trained Men Ambrotose AO^® ^Pre**	**Trained Men Ambrotose AO^® ^Post**	**Trained Men Placebo Pre**	**Trained Men Placebo Post**	**Untrained Men Ambrotose AO^® ^Pre**	**Untrained Men Ambrotose AO^® ^Post**	**Untrained Men Placebo Pre**	**Untrained Men Placebo Post**

Cholesterol (mg·dL^-1^)†	155.7 ± 9.7	149.0 ± 5.4	160.7 ± 12.1	159.9 ± 9.6	173.0 ± 13.6	169.8 ± 16.4	171.2 ± 13.6	173.6 ± 16.1
Triglycerides (mg·dL^-1^)*	82.3 ± 11.6	93.6 ± 16.4	98.7 ± 17.7	95.3 ± 14.5	103.6 ± 31.6	80.2 ± 16.8	69.2 ± 10.6	94.0 ± 32.3
HDL-C (mg·dL^-1^)*	44.7 ± 2.9	45.1 ± 3.5	45.2 ± 3.0	47.0 ± 3.6	51.2 ± 6.1	53.0 ± 3.8	51.8 ± 4.0	46.2 ± 5.7
VLDL-C (mg·dL^-1^)*	16.6 ± 2.3	18.6 ± 3.2	19.7 ± 3.5	19.1 ± 2.8	20.8 ± 6.4	16.0 ± 3.4	14.0 ± 2.0	18.8 ± 6.5
LDL-C (mg·dL^-1^)†*	94.4 ± 8.5	85.3 ± 3.9	95.6 ± 9.8	93.7 ± 8.0	101.0 ± 13.2	100.8 ± 15.4	105.4 ± 13.7	108.6 ± 16.2
LDL/HDL*	2.2 ± 0.3	2.0 ± 0.2	2.2 ± 0.3	2.1 ± 0.3	2.2 ± 0.6	2.0 ± 0.4	2.1 ± 0.5	2.7 ± 0.7

B								

**Variable**	**Trained Women Ambrotose AO^® ^Pre**	**Trained Women Ambrotose AO^® ^Post**	**Trained Women Placebo Pre**	**Trained Women Placebo Post**	**Untrained Women Ambrotose AO^® ^Pre**	**Untrained Women Ambrotose AO^® ^Post**	**Untrained Women Placebo Pre**	**Untrained Women Placebo Post**
Cholesterol (mg·dL^-1^)†	153.0 ± 5.8	146.7 ± 12.6	160.0 ± 14.2	154.7 ± 11.4	169.7 ± 9.4	167.7 ± 10.1	174.0 ± 6.4	175.3 ± 7.3
Triglycerides (mg·dL^-1^)*	56.0 ± 6.9	52.1 ± 6.1	69.1 ± 11.2	66.3 ± 10.5	66.0 ± 8.6	70.3 ± 20.6	63.0 ± 11.4	71.5 ± 20.8
HDL-C (mg·dL^-1^)*	65.1 ± 4.3	63.1 ± 6.7	65.1 ± 7.6	61.7 ± 6.7	60.9 ± 3.5	62.8 ± 5.6	64.3 ± 6.1	69.2 ± 6.3
VLDL-C (mg·dL^-1^)*	11.3 ± 1.4	10.4 ± 1.2	14.0 ± 2.3	13.3 ± 2.2	13.1 ± 1.8	14.0 ± 4.1	12.8 ± 2.3	14.2 ± 4.1
LDL-C (mg·dL^-1^)†*	76.6 ± 5.5	73.1 ± 6.1	80.9 ± 6.9	79.7 ± 6.4	95.7 ± 9.0	90.8 ± 9.3	96.8 ± 7.9	92.0 ± 8.7
LDL/HDL*	1.2 ± 0.1	1.2 ± 0.1	1.3 ± 0.1	1.3 ± 0.1	1.6 ± 0.1	1.5 ± 0.2	1.6 ± 0.2	1.4 ± 0.2

Values are mean ± SEM.

† Training status effect: Cholesterol (p = 0.009); LDL-C (p = 0.009)

* Sex effect: Triglycerides (p = 0.0005); HDL-C (p < 0.0001); VLDL-C (p = 0.0006); LDL-C (p = 0.002); LDL/HDL (p < 0.001)

No other statistically significant differences were noted (p > 0.05).

### Dietary Data

No difference was noted between conditions in subjects' dietary intake for total kilocalories, grams of protein, carbohydrate, or fat, or for vitamin C, vitamin E, or vitamin A intake (p > 0.05). However, other than vitamin E, trained subjects consumed significantly more of each nutrient category than untrained subjects and men consumed significantly more than women (p < 0.05). Data are presented in Table 6.

**Table 6:** Dietary data of men (A) and women (B) during the seven days before exercise testing following three weeks of supplementation with Ambrotose AO^® ^at a dosage of 4 capsules per day and placebo (cross-over design with a three week washout between conditions).

A				
**Variable**	**Trained Men Ambrotose AO^®^**	**Trained Men Placebo**	**Untrained Men Ambrotose AO^®^**	**Untrained Men Placebo**

Kilocalories**	2463 ± 68	2764 ± 202	1958 ± 373	1880 ± 331
Protein (g)†*	107 ± 9	123 ± 8	88 ± 20	98 ± 29
Carbohydrate (g)**	329 ± 22	362 ± 35	234 ± 58	197 ± 55
Fat (g)*	85 ± 8	94 ± 11	64 ± 12	68 ± 9
Vitamin C (mg)†*	253 ± 108	193 ± 48	83 ± 38	90 ± 31
Vitamin E (mg)	7 ± 2	8 ± 1	16 ± 13	9 ± 4
Vitamin A (RE)*	10499 ± 3377	7488 ± 1847	4894 ± 1068	8030 ± 2768

B				

**Variable**	**Trained Women Ambrotose AO^®^**	**Trained Women Placebo**	**Untrained Women Ambrotose AO^®^**	**Untrained Women Placebo**

Kilocalories**	1589 ± 99	1368 ± 143	1289 ± 153	1478 ± 179
Protein (g)†*	89 ± 16	79 ± 17	54 ± 9	66 ± 9
Carbohydrate (g)**	177 ± 21	156 ± 19	160 ± 16	176 ± 13
Fat (g)*	55 ± 5	44 ± 4	48 ± 10	58 ± 13
Vitamin C (mg)†*	89 ± 31	58 ± 13	39 ± 11	64 ± 14
Vitamin E (mg)	6 ± 2	6 ± 2	3 ± 1	4 ± 2
Vitamin A (RE)*	4118 ± 926	5494 ± 1685	2561 ± 652	2440 ± 537

Values are mean ± SEM.

† Training status effect: Protein (p = 0.05); Vitamin C (p = 0.03)

* Sex effect: Protein (p = 0.007); Fat (p = 0.0006); Vitamin C (p = 0.01); Vitamin A (p = 0.006)

** Sex × training status effect: Kilocalories (p = 0.04); Carbohydrate (p < 0.002);

No other statistically significant differences were noted (p > 0.05).

### Antioxidant Capacity and Oxidative Stress Biomarker Data: Resting

**Figure 2:**
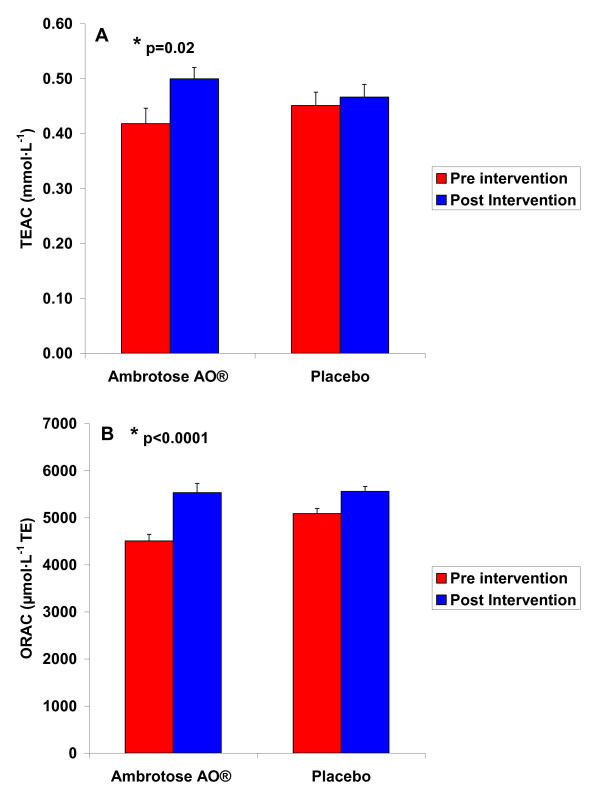
**Serum Trolox Equivalent Antioxidant Capacity (TEAC) and Oxygen Radical Absorbance Capacity (ORAC) of 25 subjects (12 men and 13 women) before and following three weeks of supplementation with Ambrotose AO^® ^at a dosage of 4 capsules per day and placebo (cross-over design with a three week washout between conditions)**. Values are mean ± SEM. For TEAC: Condition × time interaction (p = 0.17) * Paired contrast between pre and post intervention for Ambrotose AO^® ^(p = 0.02) For ORAC: Condition × time interaction (p = 0.01) * Paired contrast between pre and post intervention for Ambrotose AO^® ^(p < 0.0001)

With regards to the pre-post intervention comparison of Ambrotose AO^® ^and placebo in resting blood samples, the findings were as follows: For TEAC, a sex × training status × condition effect was noted (p = 0.009), with trained men having the highest TEAC with the Ambrotose AO^® ^condition. A sex effect was also noted (p < 0.0001), with men having higher values than women. A time effect was also noted (p = 0.01), with values higher post intervention compared to pre intervention. No other effects were noted for TEAC (p > 0.05). Although the condition × time interaction effect was not significant (p = 0.17), contrast analysis indicated that TEAC was higher post intervention compared to pre intervention for the Ambrotose AO^® ^condition (p = 0.02; Cohen's *d *= 0.63). Data are presented in Figure 2A.

For ORAC, a sex effect (p = 0.003; women having higher values than men), a time effect (p = 0.04; post intervention higher than pre intervention), a training status effect (p = 0.0002; trained subjects higher than untrained), and a condition effect (p = 0.008; placebo higher than Ambrotose AO^®^) was noted. A sex × training status effect was noted (p < 0.0001), with trained women higher than all other groups of participants. A condition × time effect was also noted (p = 0.01), with ORAC increasing more from pre to post intervention with Ambrotose AO^® ^than with placebo. Contrast analysis indicated that ORAC was higher post intervention compared to pre intervention for the Ambrotose AO^® ^condition (p < 0.0001; Cohen's *d *= 1.67). No other effects were noted for ORAC (p > 0.05). Data are presented in Figure 2B.

**Figure 3:**
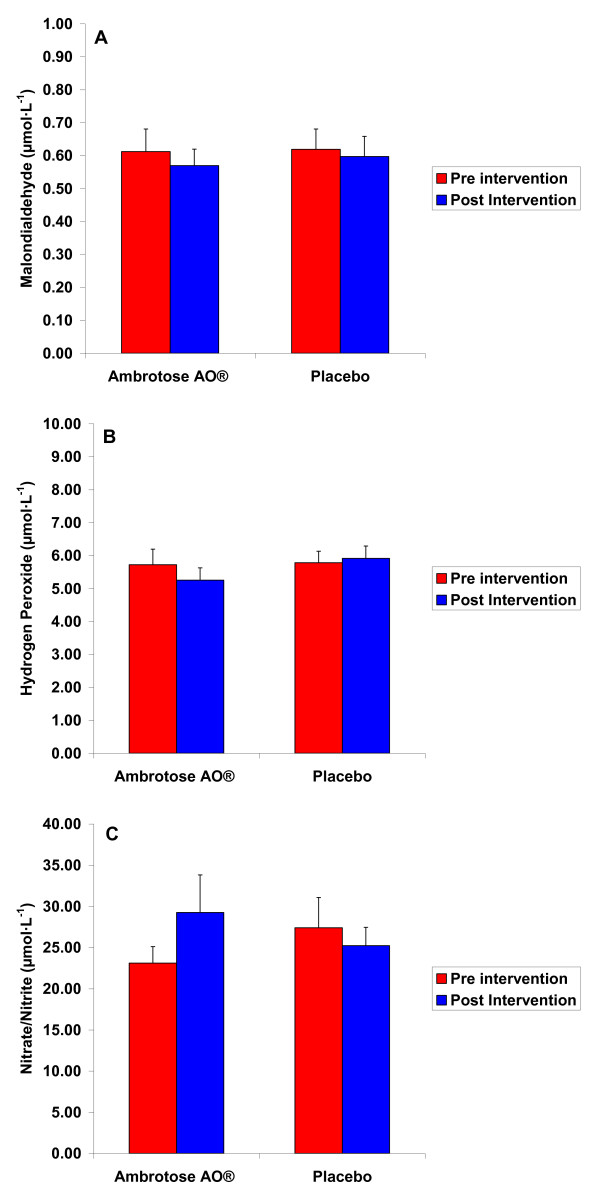
**Plasma Malondialdehyde (MDA), Hydrogen Peroxide (H_2_O_2_), and Nitrate/Nitrite (NOx) of 25 subjects (12 men and 13 women) before and following three weeks of supplementation with Ambrotose AO^® ^at a dosage of 4 capsules per day and placebo (cross-over design with a three week washout between conditions)**. Values are mean ± SEM. For MDA: Condition × time interaction (p = 0.77) Paired contrast between pre and post intervention for Ambrotose AO^® ^(p = 0.61) For H_2_O_2_: Condition × time interaction (p = 0.53) Paired contrast between pre and post intervention for Ambrotose AO^® ^(p = 0.41) For NOx: Condition × time interaction (p = 0.11) Paired contrast between pre and post intervention for Ambrotose AO^® ^(p = 0.12)

For MDA, a sex effect was noted (p < 0.0001), with men having higher values than women. No other effects were noted for MDA (p > 0.05). Data are presented in Figure 3A.

For H_2_O_2_, a sex effect was noted (p = 0.03), with men having higher values than women. No other effects were noted for H_2_O_2 _(p > 0.05). Data are presented in Figure 3B.

For NOx, no significant effects were noted (p > 0.05), although the condition × time interaction *approached *statistical significance (p = 0.11). Contrast analysis indicated a trend for higher NOx post intervention compared to pre intervention for the Ambrotose AO^® ^condition (p = 0.12; Cohen's *d *= 0.49). Data are presented in Figure 3C.

### Antioxidant Capacity and Oxidative Stress Biomarker Data: Exercise-Induced

**Figure 4:**
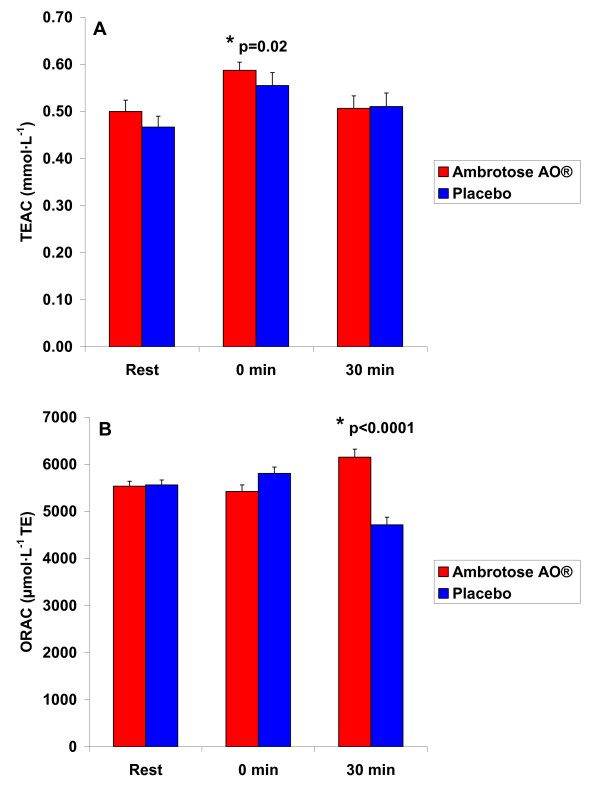
**Serum Trolox Equivalent Antioxidant Capacity (TEAC) and Oxygen Radical Absorbance Capacity (ORAC) of 25 subjects (12 men and 13 women) before and at 0 and 30 minutes after a graded exercise treadmill test to exhaustion, before and following three weeks of supplementation with Ambrotose AO^® ^at a dosage of 4 capsules per day and placebo (cross-over design with a three week washout between conditions)**. Values are mean ± SEM. For TEAC: *Time effect (p = 0.02) For ORAC: Condition × time interaction (p < 0.0001) * Paired contrast between Ambrotose AO^® ^and placebo (p < 0.0001)

With regards to the pre-post intervention comparison of Ambrotose AO^® ^and placebo in blood samples collected before and after acute exercise, the findings were as follows: For TEAC, a sex effect was noted (p < 0.0001), with men having higher values than women. A time effect was also noted (p = 0.02), with values higher at 0 minutes post exercise compared to rest (pre exercise). Data are presented in Figure 4A. No other effects were noted for TEAC (p > 0.05), although the training status effect approached statistical significance (p = 0.09), with trained subjects having higher values than untrained subjects.

For ORAC, a sex effect (p = 0.01; women having higher values than men), a training status effect (p < 0.0001; trained subjects higher than untrained), and a condition effect (p = 0.0005; Ambrotose AO^® ^higher than placebo) was noted. A sex × training status effect was noted (p < 0.0001), with trained women higher than all other groups of participants. A condition × time effect was also noted (p < 0.0001), with ORAC higher at 30 minutes post exercise for Ambrotose AO^® ^as compared to placebo. No other effects were noted for ORAC (p > 0.05). Data are presented in Figure 4B.

**Figure 5:**
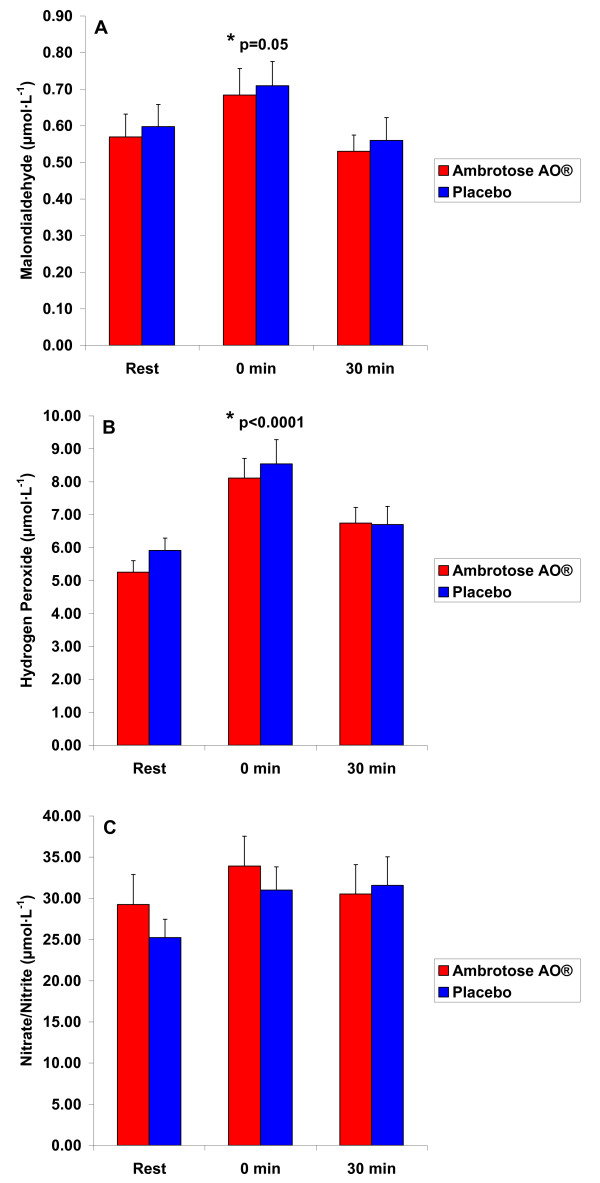
**Plasma Malondialdehyde (MDA), Hydrogen Peroxide (H**_**2**_**O**_**2**_**), and Nitrate/Nitrite (NOx) of 25 subjects (12 men and 13 women) before and at 0 and 30 minutes after a graded exercise treadmill test to exhaustion, before and following three weeks of supplementation with Ambrotose AO^® ^at a dosage of 4 capsules per day and placebo (cross-over design with a three week washout between conditions)**. Values are mean ± SEM. For MDA: *Time effect (p = 0.05) For H_2_O_2_: *Time effect (p < 0.0001)

For MDA, a sex effect was noted (p < 0.0001), with men having higher values than women. A time effect was also noted (p = 0.05), with values higher at 0 minutes post exercise compared to rest (pre exercise). Data are presented in Figure 5A. No other effects were noted for MDA (p > 0.05).

For H_2_O_2_, a sex effect was noted (p = 0.007), with men having higher values than women. A time effect was also noted (p < 0.0001), with values higher at 0 minutes post exercise compared to rest (pre exercise). Data are presented in Figure 5B. No other effects were noted for H_2_O_2 _(p > 0.05).

For NOx, no significant effects were noted (p > 0.05), although the time effect *approached *statistical significance (p = 0.13). Data are presented in Figure 5C.

## Discussion

Findings from the present investigation indicate that Ambrotose AO^® ^supplementation at a dosage of 4 capsules per day given to young, healthy, exercise trained and untrained men and women increased resting blood antioxidant capacity and appeared to be well-tolerated and safe, based on subject reporting in addition to complete blood count, metabolic, and lipid panel data. The supplement also enhanced the 30 minute post exercise antioxidant capacity of blood, as measured by serum ORAC. However, no statistically detected difference was observed in resting or exercise-induced oxidative stress biomarkers, physical or mental quality of life, or exercise time to exhaustion. In comparing to recent literature, some expected differences between men and women [23,24], as well as between trained and untrained subjects [23], were also observed.

The data presented above from a controlled, double-blind research study are compatible with the results in previous preliminary open-label studies which support the use of Ambrotose AO^® ^as an antioxidant supplement. Based on these findings, we accept our hypothesis that Ambrotose AO^® ^would increase resting antioxidant capacity, but reject our hypothesis that we would note a decrease in oxidative stress biomarkers. Additionally, we accept our hypothesis that acute exercise would result in an increase in oxidative stress in both conditions, but reject our hypothesis that attenuation would be observed with Ambrotose AO^® ^treatment (with the exception of a higher ORAC value at 30 minutes post exercise).

The changes noted in blood antioxidant capacity from pre to post intervention are similar to, albeit slightly less than, those reported in the previous two open-label designs using Ambrotose AO^® ^[15,16]. Moreover, our increases of approximately 22% in ORAC and 19% in TEAC are similar to other previously published work using either whole foods or antioxidant supplements. For example, an increase in serum ORAC has been documented following ingestion of strawberries (14.4%) and spinach (28.5%) [25], buckwheat honey (7%) [26], and concord grape juice (8%) [27]. In contrast, ingestion of a high-carotenoid content diet had no effect on serum ORAC [28]. The results of dietary supplementation trials on serum ORAC have been mixed. For example, in a placebo-controlled trial of healthy adults, a single 100 g dose of wild blueberry powder significantly increased serum ORAC by up to 16% [29] and a single relatively high (1.25 g) dose of vitamin C increased serum ORAC by 23% [25]. In a second placebo-controlled study of 500 mg/day vitamin C, serum ORAC was noted to be significantly increased, (2.5%) [30]. In contrast to these findings, other supplementation studies did not show any effect on serum ORAC: an antioxidant supplement (vitamin E, beta-carotene, ascorbic acid, selenium, alpha-lipoic acid, N-acetyl 1-cysteine, catechin, lutein, and lycopene) [31]; either of two antioxidant supplements (an antioxidant vitamin/mineral tablet or a vitamin/mineral/fruit and vegetable powder capsule) [28]; or a fruit-based antioxidant drink [32]. Clearly, data are mixed with regards to dietary supplements to increase blood antioxidant status. Discrepancies may be related to the health status of the subject population, the subjects' starting antioxidant status, the bioavailability of the supplement, and the time course of treatment. As with many nutritional supplements, optimal benefits of Ambrotose AO^® ^may be observed with chronic intake.

While we noted significant increases in blood antioxidant capacity with Ambrotose AO^® ^given at 2000 mg per day, it is unknown whether or not a lower dosage could provide similar effects, or whether a higher dosage could provide even more favorable effects. Moreover, while our data are in reference to a sample of young and healthy individuals, it is possible that older, deconditioned or diseased individuals might experience more robust changes in our chosen outcome measures. While we chose to include more "global" measures of antioxidant capacity, an analysis of individual enzymatic and non-enzymatic antioxidants before and after treatment with Ambrotose AO^® ^would be of interest. Lastly, while we chose to use an exercise stressor in the present study, it is possible that other stressors such as high fat or high sugar feedings may better assess the antioxidant potential of the Ambrotose AO^® ^supplement. Future research is needed to provide answers to the above questions.

We noted an increase in resting antioxidant capacity with Ambrotose AO^® ^supplementation, but no statistically detected difference was observed in resting or exercise-induced oxidative stress biomarkers between conditions. In relation to the resting data, it is possible that our lack of finding for a decrease in resting oxidative stress biomarkers with Ambrotose AO^® ^treatment is related to the relatively low initial values displayed by our subjects, similar to values we have recently reported for well-trained men and women [23]. Despite any potential antioxidant effect of the Ambrotose AO^®^, there may be little need to further decrease the already low resting levels of these oxidative stress biomarkers. This is especially true in light of the fact that a mild degree of oxidative stress, and RONS production promoting such a condition, appears a vital component of normal, healthy physiological functioning [33]. Perhaps the inclusion of individuals with higher resting oxidative stress values would allow for changes of statistical significance in relation to our chosen biomarkers.

In relation to the exercise-induced findings, our data agree with many previous reports demonstrating a small and transient increase in antioxidant capacity and oxidative stress biomarkers in response to acute aerobic exercise. We have recently presented the most comprehensive review to date on this topic, with the inclusion of over 300 original investigations [3]. In this review it is evident that acute exercise, whether aerobic or anaerobic has the *potential *to increase oxidative stress as measured in human blood samples. While this is certainly not a universal finding, most studies indicate at least a mild oxidative stress in response to acute, strenuous exercise (often of long duration) in both men and women, and in both exercise-trained and untrained individuals. Our data support these findings, evidenced by a transient increase in all measured variables (with the exception of NOx) at 0 minutes post exercise, with a rapid return towards baseline values at 30 minutes post exercise--while serum ORAC was elevated above pre exercise at this time.

With regards to the use of antioxidant supplementation in an attempt to attenuate the exercise-induced increase in oxidative stress biomarkers, several investigations have been conducted over the past two decades. The only statement that can be made with confidence at the present time is that the results are largely mixed [3,34], and are likely dependent on the type, dosage, and time frame of treatment of the antioxidant(s), the tissue sampled (e.g., skeletal muscle, blood), the exercise protocol used to induce oxidative stress, the time frame of measurement, the assays used to measure the degree of oxidative stress, the test subjects recruited (i.e., trained vs. untrained, old vs. young, healthy vs. diseased, well-nourished vs. malnourished), among other variables [19]. Detailed reviews of this topic have been presented elsewhere [35,36].

Due to these factors, and considering the individual response to antioxidant treatment, it is not surprising that we failed to note a statistically significant reduction in exercise-induced oxidative stress in the present study (although that was contrary to our initial directional hypothesis). The reality is that while certain subjects will benefit from pretreatment with antioxidant supplements for purposes of decreasing exercise-induced oxidative stress, others may not. The important point to keep in mind is that the oxidative stress response observed with moderate duration acute exercise is mild and transient. Such a response is not thought to be detrimental. To the contrary, a low grade oxidative stress appears necessary for various physiological adaptations [37]. Such a repeated exposure of the system to increased RONS production from chronic exercise training leads to an upregulation in the body's antioxidant defense system [38], thus providing adaptive protection from RONS during subsequent exercise sessions, as well as when exposed to non-exercise related conditions. In fact, exercise-induced oxidative stress may operate in a similar fashion to all other principles of exercise science. That is, in order for an adaptation to occur (e.g., increased antioxidant defense, hypertrophy, strength, etc.), the physiological stimulus applied (in this case RONS production) must exceed a certain minimal threshold, effectively overloading the system.

This above phenomenon is specific to the principle of hormesis, which states that in response to repeated exposure to various toxins and/or stressors, the body undergoes favorable adaptations that result in enhanced physiological performance and improved physical health [6,7]. Exercise-induced RONS production leads to the activation of the redox sensitive transcription factor nuclear factor (NF)-kappa (κ)B, which upon activation leads to the expression of certain antioxidant enzymes [37]. Therefore, it has been suggested recently, based on data pertaining to vitamin C supplementation in conjunction with a period of exercise training, that an attempt to minimize the post exercise increase in RONS production (via antioxidant supplementation) may actually blunt the adaptive increase in antioxidant defenses, thereby increasing an individual's susceptibility to pro-oxidant attack both at rest, as well as following subsequent exercise bouts [12,13]. This indeed merits further investigation.

Aside from blood markers of antioxidant capacity and oxidative stress, we measured quality of life using a validated questionnaire (SF-12). No significant differences were noted between Ambrotose AO^® ^and placebo conditions. Because our subjects were relatively young and healthy, all reported values for both mental and physical health that were at the top of the scoring scale prior to beginning the study. Therefore, they had little room for improvement when using the Ambrotose AO^®^, which helps to explain our lack of effect.

We also measured exercise time to exhaustion for the GXT and noted no significant difference between Ambrotose AO^® ^and placebo conditions. This lack of a physical performance effect of antioxidant supplementation agrees with previous work, which has noted little to no improvement in exercise performance following intake of antioxidants [35,39].

Finally, as a measure of safety and potential interest in relation to cardiovascular and metabolic parameters (e.g., blood lipids and glucose), we measured complete blood count, metabolic panel, and lipid panel values. No significant differences were noted between Ambrotose AO^® ^and placebo conditions for any measured variable. These finding provide safety data in relation to the short-term (3 week) intake of Ambrotose AO^® ^by young, healthy subjects.

Considering the results presented within, Ambrotose AO^® ^may prove to be an effective dietary antioxidant for purposes of improving resting blood antioxidant capacity. While our sample consisted of relatively young and healthy men and women, it is possible that more robust effects may be noted within a sample of older individuals, those with known disease, or within those with lower antioxidant capacity and higher oxidative stress due to lifestyle factors (e.g., cigarette smokers). Moreover, while we used an exercise stressor in the present study in an attempt to increase RONS and to test the antioxidant potential of Ambrotose AO^®^, other stressors such as the ingestion of excess saturated fat or high glycemic carbohydrate feedings may better assess the antioxidant potential of the Ambrotose AO^® ^supplement. Future research is needed to provide answers to the above questions.

## Conclusion

The findings presented here indicate that Ambrotose AO^® ^may improve resting blood antioxidant capacity and may enhance post exercise blood antioxidant capacity in young, exercise trained and untrained men and women. However, this supplement does not appear necessary for purposes of decreasing exercise-induced oxidative stress within a sample of young, healthy men and women.

## Competing interests

Financial support for this work was provided by Mannatech, Incorporated (Coppell, TX). The investigators and the University of Memphis have no direct or indirect interest in Ambrotose AO^® ^or Mannatech, Incorporated.

## Authors' contributions

RJB was responsible for the study design, biochemical work, statistical analyses, and writing of the final report. REC, MMB, and KHFW were responsible for subject recruitment, screening, and retention, data collection and entry, and blood collection and processing. KHFW and REC were also responsible for assisting with biochemical work and manuscript preparation. All authors read and approved the manuscript.
